# Nano Co-Crystal Embedded Stimuli-Responsive Hydrogels: A Potential Approach to Treat HIV/AIDS

**DOI:** 10.3390/pharmaceutics13020127

**Published:** 2021-01-20

**Authors:** Bwalya A. Witika, Jessé-Clint Stander, Vincent J. Smith, Roderick B. Walker

**Affiliations:** 1 Division of Pharmaceutics, Faculty of Pharmacy, Rhodes University, Makhanda 6140, South Africa; bwitika@ddtcollegeofmedicine.com; 2 Department of Chemistry, Faculty of Science, Rhodes University, Makhanda 6140, South Africa; g16s4830@campus.ru.ac.za (J.-C.S.); v.smith@ru.ac.za (V.J.S.)

**Keywords:** lamivudine, zidovudine, nano co-crystals, stimuli-responsive hydrogel, modified drug release

## Abstract

Currently, the human immunodeficiency virus (HIV) that causes acquired immunodeficiency syndrome (AIDS) can only be treated successfully, using combination antiretroviral (ARV) therapy. Lamivudine (3TC) and zidovudine (AZT), two compounds used for the treatment of HIV and prevention of disease progression to AIDS are used in such combinations. Successful therapy with 3TC and AZT requires frequent dosing that may lead to reduced adherence, resistance and consequently treatment failure. Improved toxicity profiles of 3TC and AZT were observed when combined as a nano co-crystal (NCC). The use of stimuli-responsive delivery systems provides an opportunity to overcome the challenge of frequent dosing, by controlling and/or sustaining delivery of drugs. Preliminary studies undertaken to identify a suitable composition for a stimulus-responsive in situ forming hydrogel carrier for 3TC-AZT NCC were conducted, and the gelation and erosion time were determined. A 25% *w/w* Pluronic^®^ F-127 thermoresponsive hydrogel was identified as a suitable carrier as it exhibited a gelation time of 5 min and an erosion time of 7 days. NCC-loaded hydrogels were evaluated using in vitro dissolution and cytotoxicity assays. In vitro dissolution undertaken using membrane-less diffusion over 168 h revealed that 3TC and AZT release from NCC-loaded hydrogels was complete and followed zero-order kinetic processes, whereas those loaded with the micro co-crystal and physical mixture were incomplete and best described using the Korsmeyer–Peppas kinetic model. The release of AZT and 3TC from the physical mixture and MCC-loaded gel exhibited a value for *n* of 0.595 for AZT release from the physical mixture and 0.540 for the MCC technology, whereas the release exponent for 3TC was 0.513 for the physical mixture and 0.557 for the MCC technology indicating that diffusion and erosion controlled 3TC and AZT release. In vitro cytotoxicity assay data revealed that the addition of NCC to the thermoresponsive hydrogel resulted in an improved cell viability of 88.0% ± 5.0% when compared to the cell viability of the NCC of 76.9% ± 5.0%. The results suggest that the use of a thermoresponsive nanosuspension may have the potential to be delivered as an intramuscular injection that can subsequently increase bioavailability and permit dose reduction and/or permit use of a longer dosing frequency.

## 1. Introduction

The treatment of HIV and AIDS has improved since the advent of potent combination antiretroviral therapy (ART), which has dramatically reduced HIV-associated morbidity and mortality and transformed the treatment of HIV into a manageable chronic condition resulting in a life expectancy approaching that of uninfected individuals [1,2]. ART is also effective for the prevention of sexual transmission of HIV in patients who present with adequately suppressed viral loads. Lamivudine (3TC) and zidovudine (AZT) are two nucleosides/nucleotide reverse transcriptase inhibitors (NRTI) used in combination to achieve viral suppression [3,4,5]. NRTI are analogues of endogenous 2-deoxy-nucleosides and -nucleotides that are inactive in the parent form requiring phosphorylation by host cell kinase and phosphotransferase enzymes to form deoxynucleoside triphosphate (dNTP) analogues, which precipitate viral inhibition by competing with endogenous dNTP during incorporation in reverse transcriptase (RT) in the virus, resulting in termination of the viral reverse transcripts and inhibition of the critical pro-viral DNA synthesis step, prior to integration into the host cell genome [3]. Due to frequent dosing requirements, low bioavailability, toxicity including bone marrow suppression, gastrointestinal disturbances, skeletal muscle and cardiomyopathy, mitochondrial aberrations such as lipoatrophy, lactic acidosis and hepatic steatosis have limited the successful application of ARV therapy [6,7,8]. The physicochemical properties of each API are summarized in Table 1.

Parenteral delivery of active pharmaceutical ingredients (API) in human and veterinary medicine makes use of a range of biocompatible materials, which, in many cases, are intended to release the API over a prolonged period in a manner to prevent the occurrence of harmful side effects. In these systems, the biological environment and design features of the dosage form may determine the rate and extent of release of API from the technology [23,24,25,26].

Approaches for the production of long-acting technologies include loading API into a carrier vehicle such as microparticles or implants or structural modification of the API to alter hydrophilicity or reduce the rate of elimination [27].

Extended-release parenteral delivery systems range from aqueous suspensions that prolong release as a consequence of slow dissolution of the API at the site of administration to more complex technologies that are biodegradable polymeric nano-/microparticles or implants that may gel, in situ [28,29,30,31].

The intramuscular site of administration is accessed by injecting the dose through the skin into the underlying muscle tissue. The structure of the musculoskeletal system is such that blood vessels extend into muscle tissues and surround each fibre with several capillaries [32]. The perfusion of muscle tissue with blood is necessary for the successful distribution of O_2_ and nutrients to muscle cells, removal of metabolic by-products, thereby making it a useful site of administration for systemic delivery.

To understand the potential effects of process and formulation parameters on extended-release parenteral delivery product performance, extensive in vitro characterization must be undertaken. However, in vitro methods used to monitor the release of an API from novel release parenteral delivery systems, seldom mimic the in vivo environment adequately [33,34]. Extended-release intramuscular and subcutaneous products are retained at the site of administration and are designed to release API slowly with the ultimate goal of achieving constant plasma concentrations. The biological environment has a significant impact on in vivo release therefore, understanding how an API is released in vivo, is essential for the optimization of in vivo performance. In situ gelation of intramuscularly administered materials can be triggered by the in vivo environment such as temperature, pH and ionic concentration [35,36,37,38], and the use of environment and/or stimuli-responsive hydrogels to achieve sustained release to increase the duration of action of an API offers a unique approach for the delivery of antiretrovirals (ARV). Ideally, these systems would be an injectable free-flowing liquid solution at ambient temperature that undergoes gelation under physiological conditions with minimal syneresis. Furthermore, addition of the payload should be easily achieved by simple mixing [39]. When administered parenterally, these systems should exhibit a pH close to neutrality and should be bioresorbable. The biomaterials to be used in these systems must exhibit biocompatibility to ensure safety [40].

Pluronic^®^ F127 (PF127) is a commercially available GRAS listed hydrogel that exhibits thermoreversible activity and has been used to facilitate localized drug delivery via intramuscular, intraperitoneal and subcutaneous injection [41].

Chitosan is a polycationic biopolymer, which is soluble in acidic solution and undergoes phase separation at pH close to neutrality via deprotonation of the primary amino functional group by inorganic ions [42]. The mechanism of gelation of chitosan is a consequence of electrostatic attraction between ammonium functional groups of chitosan and inorganic ions in addition to hydrogen bonding between the chitosan chains and/or hydrophobic chitosan–chitosan interactions [43].

Natural gums including polysaccharide gums such as acacia, ghatti, tragacanth, guar and konjac and compounds derived from seaweed such as agar, alginate and carrageenans or from microorganisms such as gellan, xanthan, rhamsan gum and welan gum are relatively cheap, readily available and biocompatible and are widely used as additives in food products [44,45,46]. Natural gums and derivative thereof have been used as excipients for pharmaceutical or biomedical purposes [47] and hydrophilic matrix tabletting [48,49,50,51,52]. Gellan gum is a bacterial exopolysaccharide, which possesses a tetra-saccharide repeating unit comprising two d-glucose, one l-rhamnose and one d-glucuronic acid molecules. It is well known that gellan gum forms gels in the presence of cations due to electrostatic interaction with carboxylate groups of the saccharide polymer chains [53].

In vitro characterization of potential stimuli-responsive vehicles, namely, PF-127, chitosan and gellan gum matrices was undertaken. In vitro release of AZT and 3TC was investigated using the most suitable carrier to establish the feasibility of this approach to deliver AZT and 3TC nano co-crystals [54]. This research forms the basis for developing a technology that offers the advantages of using a 3TC/AZT nano co-crystal in an in-situ stimuli-responsive gel.

## 2. Materials and Methods

### 2.1. Materials

All reagents were of at least of analytical grade and were used without further purification. PF-127 was donated by BASF (Ludwigshafen, Germany). Low acyl gellan gum (GG) was procured from Zibo Hailan Chemical Co. Ltd. (Shandong, China). Chitosan, sodium lauryl sulphate (SLS), α-tocopheryl polyethylene glycol succinate 1000 (TPGS 1000), sodium chloride, sodium bicarbonate, potassium chloride, potassium phosphate dibasic trihydrate, magnesium chloride hexahydrate, calcium chloride, sodium sulphate and tris(hydroxymethyl) aminomethane were purchased from Merck^®^ Laboratories (Merck^®^, Wadeville, South Africa). Zidovudine and lamivudine were procured from China Skyrun Co. Ltd. (Taizhou, China). Human cervix adenocarcinoma cells (HeLa) were procured from Cellonex^®^ (Separation Scientific SA (Pty) Ltd., Pretoria, South Africa), and Dulbecco’s Modified Eagle Medium (DMEM) was acquired from Lonza Group AG (Basel, Switzerland).

HPLC-grade water was prepared in house with a RephiLe^®^ Direct-Pure UP reverse osmosis system (Microsep^®^, Johannesburg, South Africa) fitted with deionization and polishing cartridges, and the water was filtered through a 0.22 µm PES high flux capsule filter (Microsep^®^, Johannesburg, South Africa) prior to use. Honeywell Burdick and Jackson™ HPLC-grade methanol (MeOH) was purchased from Anatech Instruments (Johannesburg, South Africa).

### 2.2. Methods

#### 2.2.1. Micro Co-Crystal (MCC) and Nano Co-Crystal (NCC) Synthesis

MCCs were synthesized as previously described [55,56], and the MCC was used as a reference/control for comparison of in vitro dissolution and cytotoxicity data from experiments reported vide infra. Briefly, 1602 mg of AZT and 1374 mg of 3TC was accurately weighed using a model AG 135 Mettler Toledo (Greifensee, Switzerland) analytical balance. Then, 3TC was dissolved in 40 mL water and AZT in 15 mL ethanol (EtOH). The two solutions were mixed and stirred at 50 °C for an hour and then allowed to cool to 22 °C for 48 h to facilitate co-crystals formation.

NCCs were synthesized using a pseudo one-solvent cold-sonochemical method, as previously described [54,57]. Briefly, 1602 mg of AZT and 1374 mg of 3TC was accurately weighed using a model AG 135 Mettler Toledo (Greifensee, Switzerland) analytical balance. Then, 3TC was dissolved in 21 mL water and AZT in 18 mL methanol (MeOH) and 0.90% *w/v* SLS and 1.40% *w/v* TPGS 1000 were added to the aqueous phase. The solutions were subsequently injected rapidly into a precooled conical flask and incubated at 4 °C ± 2 °C in an ice bath while sonicating at 50 ± 6 kHz for 20 min using a Branson^®^ 8510E-MT ultrasonic bath (Danbury, CT, USA). The NCCs that formed were then harvested prior to use.

#### 2.2.2. Stimuli-Responsive Gel Carrier Synthesis

##### Thermosensitive Gel Carriers

Thermosensitive gel carriers were prepared using a cold process [58], and solution of PF-127 were prepared by weight (% *w/w*). An appropriate amount of PF-127 was placed into a 100 mL Scott Duran bottle and HPLC-grade water at 5 °C was added and the mixture was gently stirred using a Labcon^TM^ model MSH 10 magnetic stirrer (Maraisburg, South Africa) prior to placing the solution into a refrigerator set at 5 °C, until all PF-127 particles were dissolved, and a clear solution was formed.

##### Ion-Sensitive Gels

Ion-sensitive gels were prepared by accurately weighing appropriate amounts of gellan gum (GG) into 100 mL Scott Duran^®^ bottles and adding appropriate volumes of HPLC-grade water at 80 °C with gentle stirring using a Labcon^TM^ model MSH 10 magnetic stirrer (Maraisburg, South Africa) until a clear solution was formed [59].

##### Dual pH and Thermal Responsive Gels

Dual-responsive smart gels were produced using a two-step process. Initially, an appropriate amount of chitosan that would produce solutions between 1% and 2% *w/v* was dissolved in a 3% *w/v* acetic acid solution that was then used to prepare solutions of PF-127 of concentration ranging between 20% and 25% *w/w* [60].

##### Dual Ion and Thermal Responsive Gels

Dual-responsive gels were produced using a three-step process where, initially a 0.5% *w/v* GG solution was prepared by accurately weighing appropriate amounts of GG into 100 mL Scott Duran^®^ bottles and adding appropriate volumes of HPLC-grade water at 80 °C while gently stirring the mixture using a Labcon^TM^ model MSH 10 magnetic stirrer (Maraisburg, South Africa) until a clear solution had formed. A 25% *w/w* PF-127 solution was prepared by dissolving 25 g PF-127 that was accurately weighed into a 100 mL Scott Duran bottle and HPLC-grade water at 5 °C was added with gentle stirring using a Labcon^TM^ model MSH 10 magnetic stirrer (Maraisburg, South Africa). The solution was placed into a refrigerator set at 5 °C until all PF-127 particles were dissolved and a clear solution was formed. Finally, different volumes of GG and PF-127 solutions were added together and mixed and vortexed using a Scientific Industries™ model G560E Vortex Genie 2 (Johannesburg, South Africa) to produce a homogenous solution. The content of each solution tested is summarized in Table 2.

#### 2.2.3. Stimuli-Responsive Gel Screening

The gel technology was expected to exhibit a sol–gel transition time that was sufficiently long to permit administration using a syringe and needle, without solidification, but sufficiently short to minimize sheet formation following intramuscular administration. The in-house transition time specification for these studies was set at <5 min and the dosage form was intended to deliver 3TC and AZT over 7 days (168 h), and the maximum erosion time was also set to 168 h. All tests were performed on gels in the absence of API.

The dissolution medium used for assessing ion sensitivity and erosion was conventional simulated body fluid (c-SBF) prepared by accurately weighing each of the components (Table 3) in an A-grade volumetric flask. Approximately, 700 mL HPLC-grade water was added and the mixture was gently stirred until complete dissolution of the powders took place. The pH of the solution was adjusted to 7.4 using 1 M hydrochloric acid, and the final volume was adjusted to 1000 mL with HPLC-grade water [61,62].

##### Sol–Gel Transition Time

The ability of these colloidal systems to convert from a liquid, prior to exposure to a stimulus, to a semisolid or solid following exposure to that stimulus was investigated using a simple, modified inverted test tube approach to assess the sol–gel transition characteristics for each hydrogel system produced in these studies [63,64,65].

A 5 mL aliquot of each formulation was transferred into a 10.45 mm diameter test tube set in a vertical position (90° angle) in a test tube rack and placed into a model 102 Labotec water bath (Scientific Engineering, Industria North, South Africa) set at 37 °C, after which the ion-sensitive gels were immediately covered with 0.5 mL c-SBF. The test tubes containing ion-sensitive gels were gently inverted at 1-min intervals to determine the extent of gelation, whereas the thermosetting gels were rotated through 90° to determine the extent of flow, if any, of the formulation. The assessment was continued repeated up to the point a semisolid gel was formed and at that point, the sol–gel transition time was noted.

##### Erosion Time

The duration over which the test formulations remained in a gel state was determined by transferring 5 mL (*n* = 5) of each gel formulation into previously weighed test tubes and exposing the gel to the relevant stimulus until a semisolid gel was formed. The weight of gels was determined using a Mettler Toledo AG 135 analytical balance (Greifensee, Switzerland) after which the test tubes were placed in a Labotec^®^ shaking water bath (Oldham, UK) maintained at 37 °C with a Model 102 Scientific Engineering (Johannesburg, South Africa) heating element. A 0.5 mL aliquot of the c-SBF was placed onto the surface of each gel and agitated at 40 ± 10 rpm for 168 h. The c-SBF was removed at 1, 2, 6, 12, 24, 48, 72, 96, 120, 144 and 168 h and the weight of the test tubes was recorded after which the c-SBF was replaced with fresh fluid that had been maintained at 37 °C. The erosion was determined gravimetrically and the time at which complete dissolution of the gel had occurred was recorded as the erosion time.

The gel carrier identified as the most suitable for this application was further characterized using scanning electron microscopy to identify a carrier composition that did not exhibit 3TC and AZT interactions.

##### Scanning Electron Microscopy

Samples which existed as a gel at 37 °C were frozen by immersing in liquid nitrogen and subsequently dried under vacuum by lyophilization. Lyophilization was performed without cryoprotectant at −51.0 °C and 0.315 torr using a Vacutec freeze dryer (Labconco^®^ Corporation, Kansas City, Missouri, United States of America). Morphological analyses of the lyophilized gels were undertaken by field emission scanning electron microscopy using a Vega^®^ Scanning Electron Microscope (Tuscan, Czechia) at 20 kV after sputter coating with gold.

#### 2.2.4. NCC Characterization

The ZP, PS and PDI were determined using a Nano-ZS 90 Zetasizer (Malvern Instruments, Worcestershire, United Kingdom). The PS and PDI were determined using photon correlation spectroscopy (PCS) at a scattering angle of 90° at 25 °C. The samples analysed included the NCC in the mother liquor, in addition to the NCC harvested from the mother liquor redispersed in the most suitable stimuli-responsive carrier.

An aliquot (30 μL) of the each NCC dispersion was diluted with 10 mL HPLC-grade water and transferred to a 10 mm × 10 mm × 45 mm polystyrene cell, and the PS and PDI were measured immediately.

Laser Doppler Anemometry (LDA) was used to determine the ZP of the NCC in each dispersion. Dilute samples (*n* = 6) were prepared for analysis as described previously, prior to placing into folded polystyrene capillary cells, for measurement.

#### 2.2.5. High-Performance Liquid Chromatography

An in-house HPLC method for the quantitative analysis of AZT and 3TC was developed and validated using an isocratic reversed-phase HPLC separation with a Waters^®^ Alliance Model 2695 separation module equipped with a solvent delivery module, auto-sampler, online degasser and a Model 2489 PDA detector (Waters^®^, Milford, CT, USA). The data acquired were processed and reported using Waters^®^ Empower 3 software (Waters^®^, Milford, CT, USA). The stationary phase was a Phenomenex Kinetex^®^ 150 × 4.6 mm i.d. 2.6 µm C_18_ 100 Å column (Separations, Randburg, South Africa) operated at a flow rate of 0.3 mL/min using a mobile phase of acetonitrile: water in a 25:75% *v/v* ratio. The analytical run time was 10 min for all HPLC studies. Quantitation using an internal standard, hydrochlorothiazide was undertaken in the 1 and 150 μg/mL range for 3TC and 1 and 200 μg/mL range for AZT [66]. The chromatographic conditions used for the analysis are summarized in Table 4.

#### 2.2.6. In Vitro Release

In vitro release of 3TC and AZT from the test formulations was monitored using a modified membrane-less diffusion approach [67,68]. A 5 mL aliquot of each test formulation was placed into a 10.45 mm diameter test tube and mixed with 150 mg 3TC and 175 mg AZT, or a weight equivalent of the MCC or NCC using a Scientific Industries™ model G560E Vortex Genie 2 (Johannesburg, South Africa) for 5 min, to produce a system containing 30 mg/mL 3TC and 35 mg/mL AZT. The suspensions were transferred to a model 102 Labotec water bath (Scientific Engineering, Industria North, South Africa) maintained at 37 °C for a further 5 min. The weight of the test tube was recorded prior to and following loading, to establish the weight of each gel formulation tested. The test tube was returned to the water bath and 0.5 mL c-SBF was added while agitating at 40 ± 10 rpm. At intervals of 1, 2, 6, 12, 24, 48, 72, 96, 120, 144 and 168 h, all dissolution medium was removed from each test tube and the weight of the test tube was recorded, after which fresh dissolution medium was added to replace the withdrawn liquid. The release of 3TC and AZT from formulations in which the NCC produced using a bottom-up method were included was compared to the release of each compound from test formulations loaded with a physical mixture of 3TC and AZT in addition to those in which the MCC control was included. The experiments were performed in triplicate (*n* = 3) and quantitation of 3TC and AZT was undertaken using the previously validated HPLC method [66].

#### 2.2.7. Determination of Best Fit Model

To identify a model(s) that best described the in vitro release study data, 3TC and AZT release profiles were modelled using DDSolver, a Microsoft Excel add-in [69]. DDSolver offers many options for establishing the goodness of fit of a model including the correlation coefficient, coefficient determination or adjusted coefficient of determination and Akaike’s Information Criterion (AIC) amongst others [69]. In the case where the models tested use the same number of parameters, the coefficient of determination (R^2^) is suitable to identify the most appropriate model [69] and was, therefore, used in for these data.

The R^2^ generated following regression analysis of fitting 3TC and AZT release to Hixson–Crowell, zero-order, first-order, Higuchi and Korsmeyer–Peppas models was used to identify the best fit model. In all cases, the highest value for R^2^ was identified as the kinetic model that described the kinetics of 3TC and/or AZT release from NCC-, MCC- and physical mixture-loaded test formulations.

#### 2.2.8. Cytotoxicity Studies

It has been reported that the organ and species of origin of cells used for cytotoxicity assays correlate to the results observed [70,71]. As a general principle, it is important to select a suitable cell line for cytotoxicity studies which is, in part, dependent on the expected target organ(s) in vivo and drug delivery system used [72,73]. Notwithstanding, a study in which the cytotoxicity of bioactive silica nanoparticles undertaken using 19 different cell lines representing all major organ types, revealed minimal toxicity in all cell types, thereby implying that the cell line characteristics had a limited impact on the responses observed [74]. Consequently, in an attempt to assess the biocompatibility of the 3TC/AZT NCC thermoresponsive gel technology a human cell line available to us, namely, HeLa, was selected for this purpose.

HeLa cells were cultured in DMEM as previously described [54] at a temperature of 37 °C, preserved with 5% CO_2_, supplemented with foetal calf serum (10% *w/w*) and appropriate amounts of penicillin, amphotericin B and streptomycin. The HeLa cells were transferred to 96-well plates in 150 µL culture medium at a cell density of 1 × 10^4^ cells per well prior to overnight culture. The compounds, namely, MCC, NCC and a physical mixture of 3TC and AZT in aqueous media, MCC-loaded test formulation, NCC-loaded test formulation and a physical mixture of 3TC and AZT in the test formulation included in a stoichiometric ratio identical to those used to produce the NCC were all evaluated in these studies.

Moreover, 3TC and AZT were also evaluated by incubating 50 µM samples for 48 h. All cytotoxicity tests were performed in triplicate (*n* = 3). Following incubation, 20 µL 0.54 mM resazurin was added in PBS and the solution incubated for a further 2–4 h in order to establish cell viability. The number of cells surviving exposure was established using a SpectraMax^®^ M3 plate reader (Molecular Devices, San Jose, California, United States of America) to monitor the fluorescence of resorufin at an excitation wavelength of 560 nm and an emission wavelength of 590 nm. Version 5.02 GraphPad Prism software (GraphPad Holdings LLC, La Jolla, CA, USA) was used to reduce the data and identify values for IC_50_ values following nonlinear regression analysis of cell viability % vs. log (compound) data.

## 3. Results and Discussion

### 3.1. Stimuli-Responsive Carrier Screening

The gel carrier systems were assessed for sol–gel transition and erosion time following exposure to a relevant stimulus. The 25% *w/w* PF 127 gels exhibited the most rapid transition time while maintaining sufficient mechanical strength in a semisolid state for 168 h. These results are summarized in Table 5.

Sol–gel transition experiments reveal that the concentration of PF-127 used has an inverse impact on time to gel, where an increase in amount of PF-127 used results in a reduction in gelation time as previously reported [75,76]. The erosion of PF-127 gels was affected by PF-127 content, where an increase in amount used resulted in gels that eroded over a longer time. It is clear that PF-127 content has a direct impact on the properties of the gels tested and this variable can be used to modulate the performance of thermosensitive mixtures to meet predefined gelation and erosion times to facilitate manufacture of a product with the desired performance characteristics.

Chitosan, a cationic pH-sensitive mucoadhesive polymer, exhibited little or no effect on the sol–gel transition time of the hydrogels more than likely due to the use of a low volume of c-SBF for evaluation of erosion and was intended to simulate the muscular environment in vivo. Consequently, the sensitivity of chitosan solutions to pH change was not observed in these studies [77]. Following addition of chitosan, the thermoresponsive behaviour of the gels suggested weakening of gel integrity and strength occurred due to disruption of the hydration sphere, presumably around the hydrophobic portion of the molecule with weakening of the gel occurring earlier than when gels manufactured using PF-127 alone were tested [60,78]. These results suggest that this dual responsive carrier composition would not be ideal for the delivery of AZT and 3TC or any other API over a 7-day period and was therefore not evaluated further.

Gels manufactured using GG exhibit swelling characteristics on exposure to external stimuli and over the 7-day test period gels absorbed dissolution medium, reflected by an increase in weight that increased as the amount of GG used in the formulation increased. Such swelling would be an undesirable feature for a depot injection as the increased volume of the gel may lead to discomfort, pain and irritation or even precipitate a prolonged inflammatory response at the site of injection in patients [27,79].

The use of different amounts of PF-127 blended with GG resulted in gels that exhibited a concentration-dependent increase in mass which was less than that observed for pure GG gels. This phenomenon was possibly due to the GG contributing only a small fraction to the overall mass of the mixture. The gels exhibited an initial increase in mass and subsequently almost complete erosion was observed by the end of the experiment. As a result of the water uptake, the use of GG in these smart gels was not considered further.

Therefore, 25% *w/w* PF-127 gels (formulation 2) were considered suitable for evaluation as a potential delivery vehicle for AZT and 3TC NCC as these were facile to manufacture and exhibited an appropriate sol–gel transition time and erosion rate.

#### PF-127 25% *w/w* Gel Characterization

**Figure 1:**
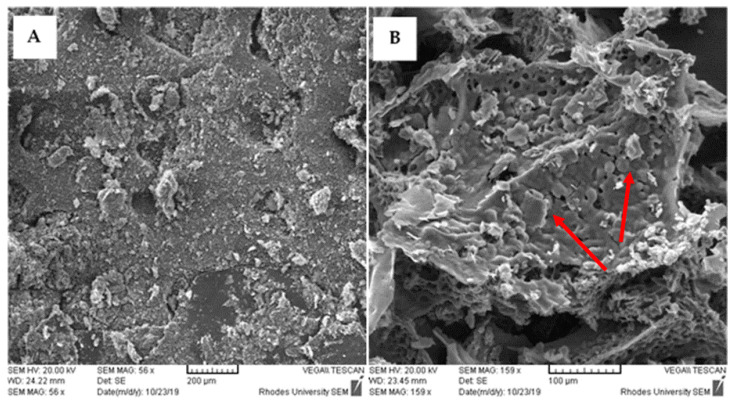
SEM images of (**A**) drug free hydrogel after lyophilization (magnification 56×) and (**B**) nano co-crystal (NCC)-loaded hydrogel/after lyophilization (magnification 159×).

The microstructural morphology of loaded and blank lyophilized gels (formulation 2) is depicted in Figure 1. The hydrogel appears as an agglomerated porous structure of irregular pore size as the removal of water resulted in the formation of an interconnected network in the hydrogel to stack, and in loaded hydrogel, the 3TC/AZT NCC are visible in the pores and pointed out with arrows.

### 3.2. Nano Co-Crystal (NCC) Characterization

NCC were successfully synthesized using a pseudo one-solvent cold-sonochemical method. The critical quality attributes (CQA) of the product in mother liquor and dispersed in a PF-127 gel are summarized in Table 6.

NCC that were dispersed in a PF-127 solution exhibited a reduction in PS and ZP. The size reduction may be due to increased solubility of the NCC in the presence of the surfactant-rich solution. Similarly, the reduction in ZP may be attributed to the presence of the additional PF-127 at the surface of the NCC, thereby reducing the overall ZP of the NCC. In addition, the possible formation of PF-127 micelles after rapid dissolution may result in entrapment of the NCC resulting in the reduction in PS and the ZP. Experiments in which attempts to determine the CQA of NCC dispersed in the PF-127 gel were unsuccessful as the gels solidified at room temperature.

### 3.3. In Vitro Release of 3TC and AZT

Nano (co)crystals and/or nanosuspensions are an effective approach for drug delivery used alone or as decorated entities that exhibit controlled and/or sustained release behaviour [80]. Furthermore in vivo activity monitoring such as tracking of materials, disease or trafficking due to enhanced permeability of nanocrystals is also possible [80]. Consequently, we attempted to control the release of 3TC and AZT from NCC in a PF-127 hydrogel in an attempt to mimic the release from microparticulate materials in a similar hydrogel that resulted in sample material retention without exhibiting 100% release of the API in that system [81].

**Figure 2:**
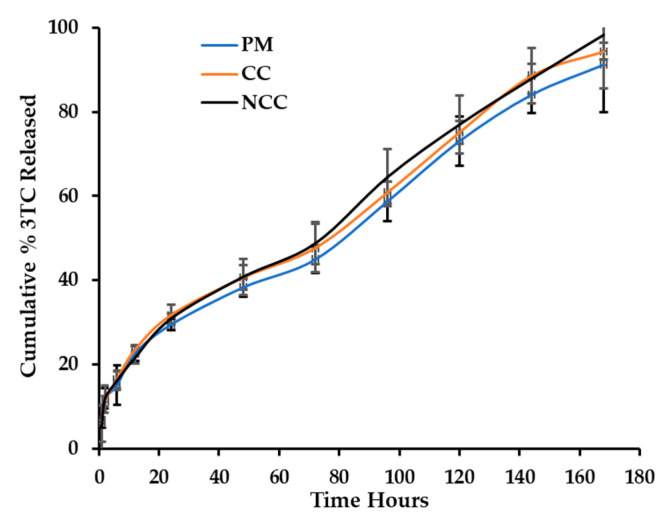
Cumulative percentage of lamivudine (3TC) released from a Pluronic^®^ F127 (PF-127) hydrogel loaded with 3TC in a physical mixture (PM), as a micro co-crystal (MCC) and nano co-crystal (NCC).

**Figure 3:**
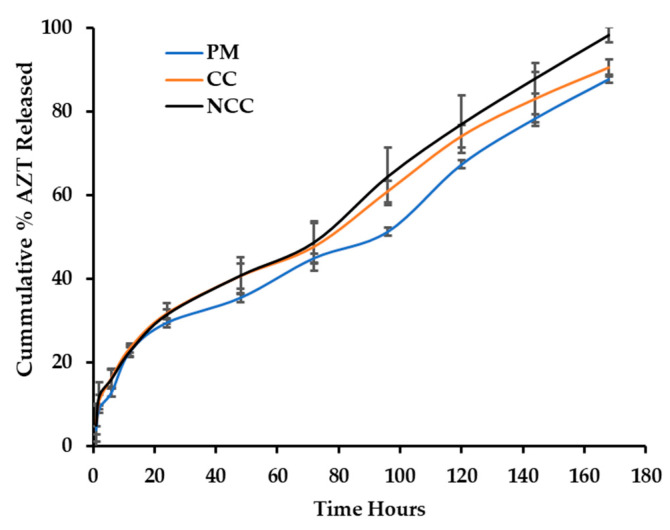
Cumulative percentage of zidovudine (AZT) released from a PF-127 hydrogel loaded with AZT in a physical mixture (PM), as micro co-crystal (MCC) and nano co-crystal (NCC).

The cumulative percentage of 3TC and AZT released from the carriers are depicted in Figure 2 (3TC) and Figure 3 (AZT).

**Figure 4:**
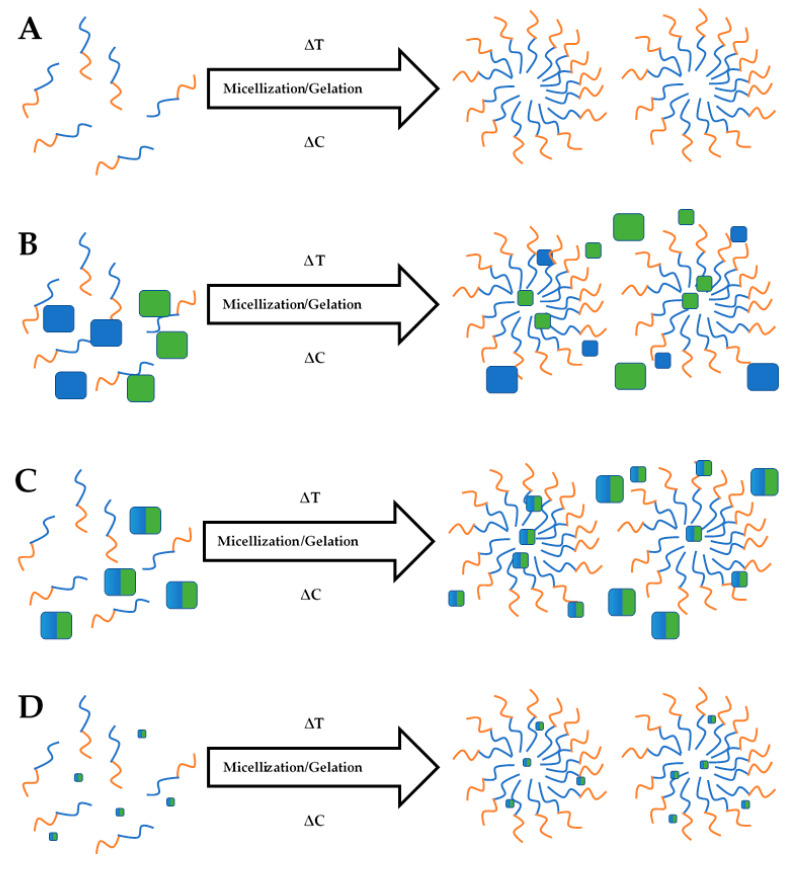
The process of micellization and subsequent gelation of the PF-127 system for control (**A**), pure active pharmaceutical ingredients (API)-loaded hydrogel (**B**), MCC-loaded hydrogel (**C**) and NCC-loaded hydrogel (**D**).

The release of 3TC and AZT from a physical mixture and MCC- and NCC-loaded hydrogel system was successfully monitored using the membraneless diffusion approach. Following exposure of the hydrogel to a relevant stimulus, it was observed that the MCC systems was, in effect, a dispersion with the presence of dissolved and undissolved materials. Zidovudine and lamivudine when included as a physical mixture or as a MCC may exhibit lower solubility than the NCC and may not be incorporated completely into micelles as the temperature of the thermosensitive system increases (Figure 4). Conversely, the NCC exhibited increased solubility and are incorporated into micelles that produced a clear semisolid gel as the temperature of the system was increased.

### 3.4. Best Fit Model

To determine the kinetics and mechanism of AZT and 3TC release from these thermoresponsive hydrogel systems, in vitro release from the hydrogels loaded with a physical mixture of 3TC and AZT, the MCC and NCC was fitted to different mathematical models using DDSolver. The resultant data are summarized in Table 7.

The coefficient of determination, R^2^, was used to identify the best fit model. The R^2^ values generated for each model suggest that all models may be used to describe 3TC and AZT release. The Higuchi model is generally used to describe release from noneroding matrices [82,83], and the gels used exhibited erosion, however, the data fitted to this model in this case. The Hixson–Crowell model can be applied to different dosage forms from which dissolution occurs in planes parallel to the surface of the drug, and the dimensions of that surface diminish proportionally such that the initial geometrical form is constant and drug release is dissolution velocity dependent and not diffusion limited [84].

It was established that 3TC and AZT release from the hydrogel and the MCC-loaded hydrogel formulations was best described using the Korsmeyer–Peppas model, whereas release from the NCC-loaded formulation was best fitted by the zero-order model and the R^2^ was >0.97 for these models.

In systems where the drug is not completely physically separated from the barrier controlling the rate of release but is homogeneously distributed in the device is monolithic, whereas when the drug is molecularly dispersed in the matrix or is rapidly and completely dissolved when water penetrates the system, monolithic solutions occur [85]. When the NCC were mixed into the PF-127 gel, a monolithic solution was formed. If a drug is homogeneously distributed within a matrix at a concentration that exceeds the solubility of the drug in the matrix, then monolithic dispersions are formed [85]. The loading of the PM and MCC into the hydrogel resulted in the formation of a monolithic dispersion.

PF-127 gel systems have been described as dissolution controlled technologies in which a combination of erosion and diffusion controlled release occur [86,87]. This is reflected by the high value for R^2^ observed when fitting data to the Korsmeyer–Peppas model. Swelling controlled the release of 3TC and AZT from these devices due to water penetrating the bulk of this device manufactured with water-soluble polymers, which also dissolve over time suggesting that erosion also has an impact on drug release from these systems [86]. However, in this case, the polymers are likely to detach from the surfaces of the solid unit of the polymer and not monomeric units on dissolution [86]. The dissolution of the Pluronic^®^ gel system occurs due to interaction of polymeric micelles with molecules of water resulting in dissociation of the micellar aggregates from the surfaces of the matrix into the dissolution medium [68].

**Figure 5:**
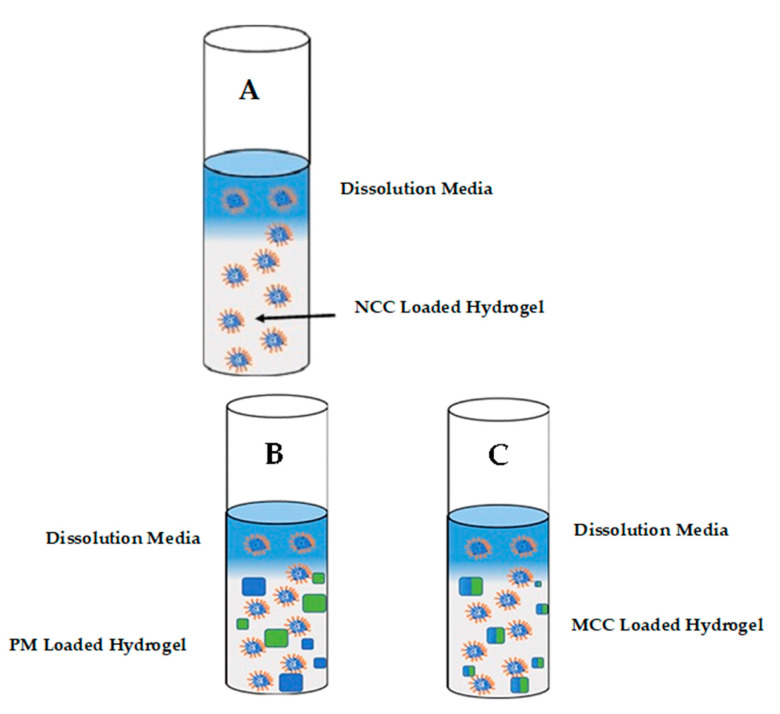
Schematic representation of 3TC and AZT release from (**A**) NCC (**B**) physical mixture (PM) and (**C**) MCC-loaded hydrogels.

AZT and 3TC release from the NCC-loaded hydrogel was best described by a zero-order kinetic process for which the release and dissolution processes are affected by dissolution of the gel. As the NCC are primarily entrapped in micelles, release from the hydrogel is influenced primarily by micellar dissociation and gel dissolution as depicted in Figure 5. If the device were prepared as a slab, as in these experiments, 3TC and AZT release would follow an approximate zero-order kinetic process as each time interval corresponds to the dissolution of a layer of the PF-127 gel with subsequent release of the 3TC and AZT in that layer [88].

These findings are similar to those observed for other systems in which monolithic solutions were evaluated. Specifically paclitaxel nanocrystals were incorporated into PF-127 micelles in a hydrogel system, from which paclitaxel release was dependent on surface area and rate of erosion of carrier gel [89]. The intratumoral injectable formulation exhibited zero-order release kinetics in vitro from a nanocrystal-loaded hydrogel [89]. Similarly, a controlled-release melanotan-I (MT-I) formulation in PF-127 exhibited zero-order in vitro release of the MT-I from the PF-127 gel carrier. These experiments were monitored using a membraneless diffusion in vitro model to evaluate the behaviour of the API and gel simultaneously. PF-127 gels exist with large numbers of micelles and aqueous channels through which the solute was able to diffuse to be released, in addition to the dissolution of the gel [90]. In another study, propranolol hydrochloride, metronidazole and cephalexin release was monitored using different temperatures, PF-127 content, drug loading and stirring speeds and revealed that release followed a zero-order kinetic process that was primarily controlled by gel dissolution for all compounds [91].

The release of AZT and 3TC from the physical mixture and MCC-loaded PF127 monolithic dispersion was best described by the Korsmeyer–Peppas model with an exponent, *n*, for AZT release of 0.595 from the physical mixture and 0.540 from the MCC technology, whereas for 3TC, the release exponent was 0.513 for the physical mixture and 0.557 for the MCC suspension (Table 7). The geometry of this dosage form was, in this case, assumed to be a slab as only one surface was exposed to the dissolution medium during testing. The phenomenon of macromolecule relaxation occurs at the glassy-rubbery interface and the extent to which this affects release of an API from any delivery system can be inferred from the value of *n*. The mechanism of 3TC and AZT release for both the MCC and the physical mixture occurred via an anomalous process in which diffusion and erosion contributed to release from these gel formulations. The controlling mechanism of release from hydrophilic matrices is the inward flux of the dissolution medium into the core of the delivery system, which causes swelling and subsequent dissolution of the polymeric system used to build the matrix and where, during the initial stages of swelling of the hydrogel, drug diffusion takes place from the inner glassy phase or the outer swollen rubbery phase [92].

When using a solution of API to form a gel using a cold addition approach, the resultant system is usually a molecular dispersion in which the drug is homogenously dispersed throughout the hydrogel, i.e., a monolithic system. However, on physical/direct addition of each API to the polymer solution with subsequent agitation, a milky suspension or monolithic dispersion was produced. The inclusion of 3TC, AZT and the MCC may not have resulted in the formation of a homogenously distributed solution in the polymeric matrix prior exposure to a stimulus, resulting in only some of the 3TC and/or AZT ultimately being dissolved in the hydrogel, which was then subsequently taken up into micelles and only released upon dissolution of the hydrogel, as depicted in Figure 5B,C. The undissolved 3TC and/or AZT are released following dissolution of the gel and/or by diffusion through the microporous structure of the intact hydrogel albeit to a lesser extent [93]. The suspension likely forms a barrier between the MCC and the receptor medium at the point at which 3TC and AZT are released, which may limit the release of these compounds by one of two mechanisms, namely, the reduction in dissolution medium penetrating the polymeric matrix thereby limiting 3TC, AZT and MCC dissolution and/or matrix solvation or alternatively, diffusion of AZT and 3TC or MCC into the surrounding environment that may be limited by chemical and/or physical interaction [94].

For the MCC systems, 3TC and AZT are initially released individually or as a MCC into the PF-127 hydrogel matrix and then diffuses through and from the matrix. The porous structure of the hydrogels creates channels though which diffusion of the incorporated solute occurs resulting in Fickian diffusion-controlled transport. Polymer relaxation due to swelling of the matrix, facilitating case II transport suggested that, in this case, diffusion- and swelling-controlled release mechanisms control the release of AZT and 3TC. These results are in agreement with previously reported data in which alginate microspheres were physically mixed in PF-127 matrices prior to exposure to a stimulus [81]. In another example, while the hydrogel base may be different, chitosan microspheres loaded with levonorgestrel and embedded in a poly(vinyl alcohol) hydrogel exhibited similar release characteristics [95]. In both cases, release from the hydrogel matrix was best described by the Korsmeyer–Peppas model, which is typically applied to describing drug release from hydrophilic and porous polymer systems that adsorb dissolution medium, which, in this case, was c-SBF, and liberate incorporated API [96]. The main difference between the release of 3TC and AZT from these systems is that for the MCC, the actual dissolution of the MCC would result in the release of equimolar amounts of AZT and 3TC simultaneously, whereas the release of 3TC from the physical mixture is likely to be independent of the rate and extent of AZT dissolution.

### 3.5. In Vitro Cytotoxicity

In vitro cytotoxicity testing was undertaken to evaluate the cytotoxicity of the NCC PF-127 gel and establish whether the use of PF-127 as a carrier would improve the cytotoxicity profile of the NCC. The data generated was compared to cytotoxicity data following testing of the MCC, a physical mixture of 3TC and AZT and NCC in aqueous media [54] and revealed that the delivery of 3TC and AZT as a NCC incorporated into a PF-127 exhibits lower cytotoxicity when compared to AZT and 3TC delivered individually from a hydrogel matrix. The most likely reason for this finding is that the presence of polyethylene glycol-based TPGS 1000 in the formulation of the NCC may impart “stealth” properties to the micelles formed in the hydrogel as compared to when AZT or 3TC was included alone. However, the cytotoxicity of the NCC PF-127 hydrogel was not significantly better (*p* > 0.05) when compared to the NCC delivered from an aqueous solution [54]. None of the other combinations tested produced significant changes in cell viability with the exception of when 3TC was tested alone and where a significant reduction (*p* < 0.05) in cell viability was observed in a HeLa cell line exposed to 3TC embedded in the hydrogels in comparison to an aqueous solution of 3TC, which is likely a consequence of an increased cellular uptake of 3TC embedded in micelles.

**Figure 6:**
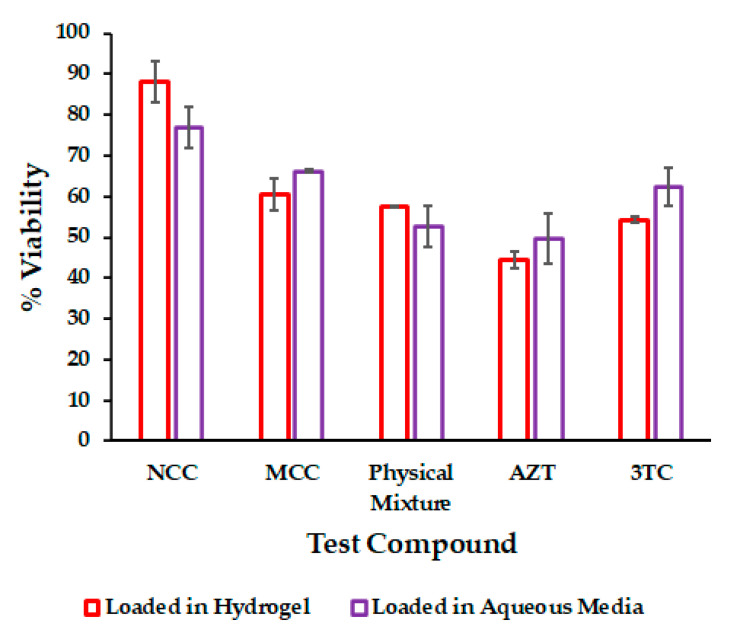
Summary of cytotoxicity data for NCC compared to 3TC and AZT alone, a physical mixture of 3TC and AZT and MCC and NCC loaded into a 25% *w/w* PF-127 hydrogel.

A summary of cytotoxicity testing results is listed in Table 8 and is illustrated graphically in Figure 6.

## 4. Conclusions

It is necessary to develop extended and controlled delivery systems for use in the treatment of chronic diseases to improve patient adherence and potentially reduce or prevent the occurrence of resistance. High doses of combination ARV are necessary for the successful treatment of HIV and/or AIDS [97], however, limited accumulation in HIV reservoir cells occurs, requiring frequent dosing that is associated with a number of undesirable adverse effects [97,98].

A PF-127 hydrogel carrier system was developed and tested, and in vitro cytotoxicity revealed that the use of a hydrogel matrix exhibited an improved cytotoxicity profile for a physical mixture of 3TC and AZT and a NCC, when compared to the use of AZT or 3TC tested alone or in combination and delivered from an aqueous solution [54].

The potential for delivering ARV compounds in a sustained manner over a pronged period as a nontoxic regimen, remains a challenge for the treatment of HIV/AIDS. The results of these studies have provided new information pertaining to a potential solution to overcome this challenge through the development and application of a combination using nano co-crystal particles delivered from an in-situ stimuli-responsive gel-forming technology.

We have demonstrated that high levels of AZT and 3TC can be incorporated into a stimulus-responsive delivery system achieved by manufacturing 3TC and AZT nano co-crystals and loading into the thermo-sensitive gel base. The use of NCC has been shown to exhibit high loading capacity with a lower associated cytotoxicity. Furthermore, the combination NCC is released from the stimulus-responsive dosage form by dissolution-control from the gel. This approach may be useful for the development of a dosage form that not only delivers HIV/AIDS therapy with macrophage and brain targeting potential but also shows a potentially improved systemic toxicity profile with the possibility of sustained delivery over a 168-h period. Despite the need for further work to assess the in vivo performance of the technology, the real clinical benefit of this work may be realized through increased adherence to HIV treatment, which may result in reduced reports of HIV resistance, fewer patients developing AIDS and, consequently, reduced occurrence of HIV-related opportunistic diseases. The potential for the NCC to target HIV reservoirs and potentially cross the blood–brain barrier (BBB) may also result in a rapid reduction in viral load counts that could reduce the potential of transmission of the virus. Furthermore, the potential to target the brain may be important for the prevention and development of AIDS dementia complex and other HIV-related neurological disorders.

## Figures and Tables

**Table 1:** Summary of the physicochemical properties of zidovudine (AZT) and lamivudine (3TC).

Properties	AZT	3TC
Chemical name	1-[(2R,4S,5S)-4-azido-5-(hydroxymethyl)tetrahydrofuran-2-yl]-5-methyl-pyrimidine-2,4(1H,3H)-dione [9]	(-)-4-amino-1-[(2R,5S)-2-(hydroxymethyl)-1,3-oxathiolan-5-yl]pyrimidin-2(1H)-one [10]
Melting point	106–112 °C (from petroleum ether); 120–122 °C (from water) [9,11,12,13]	183 °C [14]
Molecular weight (g/mol)	267.2 [9]	229.3 [10]
Chemical formula	C_10_H_13_N_5_O_4_ [9]	C_8_H_11_N_3_O_3_S [10]
Description	A white or brownish powder [9]	A white or almost white powder [10]
pK_a_ and log P	pK_a_ and log P of AZT was reported as 9.68 and 0.06, respectively [15,16]	3TC is a weak base with a pK_a_ of 4.3 by the protonation of the NH_2_ group. The log P is −1.46 [17,18,19].
BCS class	III [20]	I/III [21,22]
Solubility	Soluble in ethanol; sparingly soluble in water [9,15,16]	Soluble in water, sparingly soluble in methanol and practically insoluble in acetone [10]

**Table 2:** Composition of stimuli-responsive carriers tested.

Formulation	PF-127% *w/w*	Volume PF-127 mL	GG% *w/v*	Volume GG mL	Chitosan % *w/v*
Thermosensitive gels					
1	20	5.0	-	-	-
2	25	5.0	-	-	-
Ion-sensitive gels					
3	-	-	0.1	5.0	-
4	-	-	0.3	5.0	-
5	-	-	0.5	5.0	-
Dual thermo- and pH-sensitive gels					
6	20	5.0	-	-	1.0
7	20	5.0	-	-	2.0
8	25	5.0	-	-	1.0
9	25	5.0	-	-	2.0
Dual thermo- and ion-sensitive gels					
10	25	4.5	0.5	0.5	-
11	25	4.0	0.5	1.0	-
12	25	3.5	0.5	1.5	-

**Table 3:** Quantities used to prepare 1000 mL conventional simulated body fluid (c-SBF) adapted from [30], Elsevier, 2012.

Reagents	Concentration mg/L
Sodium chloride	7996
Sodium bicarbonate	350
Potassium chloride	224
Potassium phosphate dibasic trihydrate	228
Magnesium chloride hexahydrate	305
Calcium chloride	278
Sodium sulphate	71
Tris(hydroxymethyl) aminomethane	6057
1 M Hydrochloric acid	40 mL

**Table 4:** Chromatographic conditions for the analysis of zidovudine (AZT) and lamivudine (3TC) [66].

Parameter	Setting
Flow rate	0.3 mL/min
Injection volume	10 µL
Wavelength	266 and 271 nm
Temperature	25 °C
Mobile-phase composition	25:75% *v/v* ACN: H_2_O

**Table 5:** Sol–gel transition and erosion time for 25% *w/w* Pluronic^®^ F127 (PF 127) gels.

Formulation	Sol–Gel Transition Time Min	Erosion Time Days
**Thermosensitive gels**		
1	7	5
2	5	7
**Ion-sensitive gels**		
* 3	-	-
4	8	7
5	6	7
**Dual thermo- and pH-sensitive gels**		
6	6	5
7	6	4
8	5	6
9	5	5
**Dual thermo- and ion-sensitive gels**		
10	5	7
11	5	7
12	5	7

* Gel did not set using experimental conditions.

**Table 6:** Summary of critical quality attributes (CQA) of nano co-crystal (NCC).

CQA	Value	
	NCC	NCC in Gel
* PS (nm)	332.9 ± 42.85	243.6 ± 26.58
PDI	0.474 ± 0.040	0.495 ± 0.153
* ZP (mV)	−34.6 ± 5.56	−18.3 ± 4.45

* represent significant differences (*p* < 0.05).

**Table 7:** Results of fitting in vitro release data to mathematical models.

Formulation			Value for Kinetic Model									
	Zero-Order F = K_0_t		First-Order F = 100 × [1 − e^−k1 * t^]		Higuchi F = kHt^0.5^		Korsmeyer–Peppas F = kKPt^n^				Hixson-Crowell F = 100 × [1 − (1 − kHCt)^3^]	
	AZT	3TC	AZT	3TC	AZT	3TC	AZT	*n*	3TC	*n*	AZT	3TC
NCC	* 0.9857	* 0.9859	0.7350	0.7835	0.9801	0.9763	0.9797	0.613	0.9773	0.598	0.8868	0.8774
Physical mixture	0.9761	0.9773	0.8170	0.7522	0.9845	0.9691	* 0.9915	0.595	* 0.9783	0.513	0.8915	0.8655
MCC	0.9735	0.9786	0.7793	0.7832	0.9844	0.9832	* 0.9882	0.540	* 0.9861	0.557	0.8708	0.8739

* represent model with highest R^2^ value. *n* = release exponent.

**Table 8:** Summary of cytotoxicity results.

Compound	Viability %	
	Loaded in Hydrogel	In Aqueous Media
NCC	88.0 ± 5.0	76.9 ± 5.0
MCC	60.5 ± 3.8	66.1 ± 0.4
Physical mixture	57.5 ± 0.2	52.6 ± 5.1
AZT	44.3 ± 2.1	49.6 ± 6.2
3TC	54.2 ± 0.7	62.3 ± 4.7

## Data Availability

The data presented in this study are available on request from the corresponding author.
